# A Novel Generalization of Q-Rung Orthopair Fuzzy Aczel Alsina Aggregation Operators and Their Application in Wireless Sensor Networks

**DOI:** 10.3390/s23198105

**Published:** 2023-09-27

**Authors:** Wajid Ali, Tanzeela Shaheen, Iftikhar Ul Haq, Tmader Alballa, Alhanouf Alburaikan, Hamiden Abd El-Wahed Khalifa

**Affiliations:** 1 Department of Mathematics, Air University, PAF Complex E-9, Islamabad 44230, Pakistan; 2 Department of Mathematics, College of Sciences, Princess Nourah bint Abdulrahman University, P.O. Box 84428, Riyadh 11671, Saudi Arabia; 3 Department of Mathematics, College of Science and Arts, Qassim University, Al-Badaya 51951, Saudi Arabia or ha.ahmed@qu.edu.sa (H.A.E.-W.K.); 4 Department of Operations and Management Research, Faculty of Graduate Studies for Statistical Research, Cairo University, Giza 12613, Egypt

**Keywords:** Q-rung orthopair hesitant fuzzy sets, decision making, wireless sensor networks, optimization, Aczel–Alsina aggregation operators, efficiency

## Abstract

Q-rung orthopair fuzzy sets have been proven to be highly effective at handling uncertain data and have gained importance in decision-making processes. Torra’s hesitant fuzzy model, on the other hand, offers a more generalized approach to fuzzy sets. Both of these frameworks have demonstrated their efficiency in decision algorithms, with numerous scholars contributing established theories to this research domain. In this paper, recognizing the significance of these frameworks, we amalgamated their principles to create a novel model known as Q-rung orthopair hesitant fuzzy sets. Additionally, we undertook an exploration of Aczel–Alsina aggregation operators within this innovative context. This exploration resulted in the development of a series of aggregation operators, including Q-rung orthopair hesitant fuzzy Aczel–Alsina weighted average, Q-rung orthopair hesitant fuzzy Aczel–Alsina ordered weighted average, and Q-rung orthopair hesitant fuzzy Aczel–Alsina hybrid weighted average operators. Our research also involved a detailed analysis of the effects of two crucial parameters: λ, associated with Aczel–Alsina aggregation operators, and N, related to Q-rung orthopair hesitant fuzzy sets. These parameter variations were shown to have a profound impact on the ranking of alternatives, as visually depicted in the paper. Furthermore, we delved into the realm of Wireless Sensor Networks (WSN), a prominent and emerging network technology. Our paper comprehensively explored how our proposed model could be applied in the context of WSNs, particularly in the context of selecting the optimal gateway node, which holds significant importance for companies operating in this domain. In conclusion, we wrapped up the paper with the authors’ suggestions and a comprehensive summary of our findings.

## 1. Introduction

### 1.1. WSNs

Wireless Sensor Networks (WSNs) consist of numerous miniature, cost-effective, and self-sufficient devices known as sensor nodes. These nodes are equipped with various sensors designed to gather data from their surroundings and transmit them wirelessly. WSNs have found widespread use across a diverse range of applications, including environmental monitoring, surveillance, healthcare, industrial automation, and more. Researchers have explored this field extensively and introduced innovative models to demonstrate the practicality of WSNs in real-world scenarios. For instance, Shelebaf et al. [1] introduced a clustering model in WSNs based on the TOPSIS algorithm. Sandhu et al. [2] proposed an approach for commercial lighting control using WSNs within a multi-agent decision-making framework. Madhavi et al. [3,4] focused on fuzzy sets and their extensions, exploring their applications within wireless sensor networks (WSNs). In 2007, Pirmez et al. [5] applied fuzzy logic to the decision making in WSNs. Sreedharan et al. [6] delved into multicriteria decision making within a fuzzy environment, applying it to hybrid routing protocols for WSM. Abbassi et al. [7] developed the concept of WSNs integrated with fuzzy control systems. Kumar et al. [8] authored a paper discussing Trust-Evaluation-based Machine Learning for WSNs. Furthermore, numerous scholars have devised innovative approaches in different contexts to address real-life challenges [9,10,11,12].

### 1.2. Fuzzy Sets and Aggregation Operators

In 1965, Zadeh [13,14] introduced the concept of fuzzy sets (FSs), which have been proven to be highly effective at handling the uncertainty associated with the information within a universal set. Over time, various valuable extensions of fuzzy sets have emerged and demonstrated their advantages. These extensions include intuitionistic fuzzy sets [15,16] and hesitant fuzzy sets [17,18,19]. Ali et al. [20,21] extensively explored these fuzzy extensions within the context of three-way decision models, crafting multiple-attribute decision-making techniques applicable in practical scenarios. Alcantud et al. [22] ventured into novel rough set models built upon the foundation of extended fuzzy sets. Kamran et al. [23] devised an optimistic policy for multiple-attribute decision-making, employing a framework based on probabilistic neutrosophic hesitant fuzzy rough data. Zhang et al. [24] introduced a preference method for Pythagorean hesitant fuzzy multiple-attribute decision-making rooted in regret theory. Jin et al. [25] focused their efforts on the notion of hesitant fuzzy β neighborhoods, applying this approach to decision-making processes, particularly within the TOPSIS method. Tsai et al. [26] developed interval-valued hesitant fuzzy DEMATEL-based blockchain technology, demonstrating its utility in agricultural supply chain management, an increasingly appealing area of research. Furthermore, Mahmmod et al. [27] revisited the concept of intuitionistic hesitant fuzzy sets, consolidating their findings through similarity measures and power aggregation operators to enhance the decision-making process.

Ali et al. [28] introduced an innovative enhancement to Q-rung orthopair fuzzy sets (Q-ROFSs). Concurrently, Oraya et al. [29] integrated a multi-criterion sorting approach based on Q-rung orthopair fuzzy sets and applied this model to assess the impacts of delays in residential construction projects. Jabeen et al. [30] devised a comprehensive framework of aggregation operators to compute the information table associated with Q-rung orthopair fuzzy sets. Krishankumar et al. [31] harnessed the Q-rung framework for the selection of an IoT service provider geared towards sustainable transport in the year 2023. Additionally, Suri et al. [32] introduced an innovative framework known as the Biparametric Q-rung orthopair fuzzy entropy measure, tailored for decision-making purposes. These studies have collectively contributed to the evolving landscape of Q-ROFS research and its practical applications.

The computation of fuzzy data presents a considerable challenge. To address this issue, mathematicians have devised aggregation operators. Yager et al. [33,34,35,36,37], for instance, introduced the concepts of power average and power geometric aggregation operators, effectively applying them to tackle uncertainty in aggregation. Shi et al. [38] delved into power aggregation operators specifically designed for interval-valued intuitionistic fuzzy sets. Wei et al. [39] established power operators tailored for Pythagorean fuzzy data, while Mahmood et al. [40] analyzed operators designed for fuzzy data, applying them to decision-making tasks. In a similar vein, Bonferroni mean operators have played a pivotal role in addressing this challenge within the realm of research. Numerous scholars have explored and employed these operators within their research contexts [41,42,43,44]. One of the most influential and current aggregation operators are Aczel–Alsina operators [45]. These operators have been adapted and studied within various fuzzy environments. Senapati et al. [46] developed Aczel–Alsina operators for Pythagorean fuzzy sets, demonstrating their utility in multiple-attribute decision making. Ali et al. [47] designed Aczel–Alsina operators for p,q-quasirung orthopair fuzzy sets, applying them to decision systems. Karabacak et al. [48] extended Aczel–Alsina aggregation to handle interval neutrosophic data. In another instance, Gayen et al. [49] established a novel Aczel–Alsina triangular norm-based group decision-making approach within the context of dual hesitant q-rung orthopair fuzzy data, particularly relevant for parcel lockers’ location selection. Haq et al. [50] focused on novel fermatean fuzzy data and their interaction with Aczel–Alsina operators. Additionally, Senapati et al. [51] devised an intuitionistic fuzzy power Aczel–Alsina model to prioritize sustainable transportation-sharing practices. Feng et al. [52] constructed a method known as WASPAS, incorporating Aczel–Alsina aggregation operators to manage complex interval-valued intuitionistic fuzzy information, with applications in the domain of decision making.

### 1.3. Motivation

The literature review underscores the significance of fuzzy sets and aggregation operators, revealing their diverse applications across various domains. Recognizing this significance, we undertook the development of novel fuzzy extensions in this study. These extensions were specifically designed to address the challenges posed by uncertainty within information systems. The research presented in this paper introduced a novel concept called Q-rung orthopair hesitant fuzzy sets and explored its application in the domain of Aczel–Alsina aggregation operators. While there has been significant prior research on Q-rung orthopair fuzzy sets and hesitant fuzzy models independently, the fusion of these two frameworks, resulting in Q-rung orthopair hesitant fuzzy sets, represents a novel and unexplored territory in the field of fuzzy set theory. Additionally, the study investigated the effects of crucial parameters, λ and N, on decision-making processes, offering insights into the sensitivity of the proposed aggregation operators. Moreover, the application of this innovative model in the context of Wireless Sensor Networks (WSNs), specifically in selecting optimal gateway nodes, represents a significant research gap. The WSN application domain is of growing importance, and the incorporation of Q-rung orthopair hesitant fuzzy sets in this context will open up new avenues for enhancing the decision-making processes in WSNs, which has not been extensively explored in the prior literature. Therefore, this research addresses a gap by bridging the concepts of Q-rung orthopair fuzzy sets, hesitant fuzzy models, and their application in real-world WSN scenarios, contributing to the advancement of decision support systems in this emerging technology field. The inspiration and contributions of this paper can be summarized as follows:

- In this paper, the integration of two distinct fuzzy models, namely Q-ROFSs and HFSs, and the elucidation of their respective characteristics are introduced.
- The fundamental properties of our proposed model, offering a comprehensive exposition on monotonicity, commutativity, and boundedness, are developed.
- Within the framework of Q-ROHFSs, a set of Aczel–Alsina aggregation operators, encompassing Q-ROHFAAWA, Q-ROHFAAOWA, and Q-ROHFAAHWA, are introduced.
- A mathematical model for multiple-attribute decision making in the context of Wireless Sensor Networks (WSNs) based on our established methodology is established.
- A succinct analysis of the impact of parameter variations on alternative rankings, accompanied by graphical representations illustrating the ranking variations, is added.

**Figure 1:**
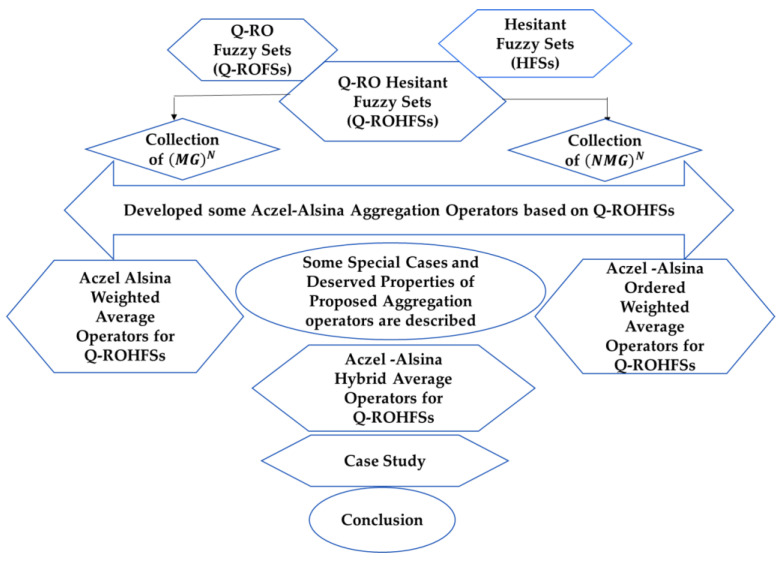
Flow chart of the paper work.

The rest of this article is divided into the subsequent portions: Section 2 introduces the fundamental notions of fuzzy extensions and aggregation operators. Section 3 outlines the integrated framework of Q-ROFSs and HFSs, along with their fundamental operations. Section 4 presents a comprehensive set of Aczel–Alsina aggregation operators tailored for Q-ROHFSs. Section 5 encompasses the algorithm used in our proposed approach and its corresponding mathematical model for Wireless Sensor Networks (WSNs). Additionally, it includes a brief discussion on the variations of parameters. Finally, the authors provide a summary of the key points and conclusions. You can refer to Figure 1 for a visual depiction of the paper’s flowchart.

## 2. Basic Concepts

In this part, we will revise some primary ideas associated with Q-rung orthopair fuzzy sets, hesitant fuzzy sets, and Aczel–Alsina aggregation operators. Table 1 explains the symbols used in the paper.

**Definition** **1** [28]**.** *For a universal set* U, *A Q-rung orthopair fuzzy set (Q-ROFS)* T *over* U *is formulated as below*.

$$T=\left(k,\gamma_{T}\left(k\right),\mu_{T}\left(k\right):k\epsilon U\right)$$

*where* $\gamma :U\to \left[0,1\right]$ *and* $\mu :U\to \left[0,1\right]$ *are, respectively, the functions granted to the grades of membership and non-membership, such that*

$$0\leq {\left(\gamma_{T}\left(k\right)\right)}^{N}+{\left(\mu_{T}\left(k\right)\right)}^{N}\leq 1,\;\;\;N\geq 1$$

*For ease of calculations, we call Q-ROFS* $T=\left(\gamma ,\mu \right)$ *with satisfying* $\gamma^{N}+\mu^{N}\leq 1$, *where* γ *and*  μ *are chosen from the unit closed interval* $\left[0,1\right]$.

**Definition** **2.** *For Q-ROFNs $T=\left(\gamma ,\mu \right),T_{1}=\left(\gamma_{1},\mu_{1}\right)$, and $T_{2}=\left(\gamma_{2},\mu_{2}\right)$, the following operators have been defined*.

(i)
$T_{1}⊕T_{2}=\left(\sqrt[{N}]{1-\left(1-\gamma_{1}^{N}\right)\left(1-\gamma_{2}^{N}\right)},\mu_{1}\mu_{2}\right),$

(ii)
$T_{1}⊗T_{2}=\left(\gamma_{1}\gamma_{2},\sqrt[{N}]{1-\left(1-\mu_{1}^{N}\right)\left(1-\mu_{2}^{N}\right)}\right),$

(iii)
$ℷT=\left(\sqrt[{N}]{1-{\left(1-\gamma^{N}\right)}^{ℷ}},\mu^{ℷ}\right)$

(iv)
$T^{ℷ}=\left(\gamma^{ℷ},\sqrt[{N}]{\left(1-{\left(1-\mu^{N}\right)}^{ℷ}\right)}\right)$

**Definition** **3.** *The score function to rank the Q-ROFN $T=\left(\gamma ,\mu \right)$ is defined as*

$$V\left(T\right)=\left(\gamma^{N}-\mu^{N}\right)$$

*In addition, an accuracy function is defined as*

$$L\left(T\right)=\left(\gamma^{N}+\mu^{N}\right)$$

*It is evident that* $-1\leq V\left(T_{i}\right)\leq 1$ and $0\leq W\left(T_{i}\right)\leq 1.$

**Definition** **4.** Let $T_{1}$ and $T_{2}$ *be two Q-ROFNs. These numbers can be compared as*,

(i) *If* $V\left(T_{1}\right)>V\left(T_{2}\right)$ *then* $T_{1}$ *is superior to* $T_{2}$ *and is represented by* $T_{1}{≻T}_{2}$.
(ii) *If* $V\left(T_{1}\right)=V\left(T_{2}\right)$, *then*
  (a) *if* $L\left(T_{1}\right)>L\left(T_{2}\right)$ *then* $T_{1}{≻T}_{2}$
  (b) *if* $L\left(T_{1}\right)=L\left(T_{2}\right)$ *then* $T_{1},T_{2}$ *are both equivalent.*

### 2.1. Hesitant Fuzzy Sets

Attansove established the idea of HFSs, which are a more powerful tool for coping with the vagueness of information.

**Definition** **5** [17]**.** *Taking U as a ground set, a HFS H over U is expressed as below*

$$H=\left(k,\alpha_{H}\left(k\right):k\epsilon U\right)$$

*where* $\alpha_{H}\left(k\right)=\left\{a_{i}\left(k\right)\right\}\left(i=1,2,..n\right)$ *and* $\alpha :U\to \left[0,1\right]$ *is a mapping dedicated to assign the collection of membership values from [0, 1]. It is necessary for a membership to satisfy the given criteria* $0\leq \alpha_{H}\left(k\right)\leq 0$.

**Definition** **6** [17]**.** *Let* $H_{1}=\alpha_{1}\left(k\right)$ *and* $H_{2}=\alpha_{2}\left(k\right)$ *be two HFNs. Then*

(i)
$H_{1}\cup H_{2}=\underset{\begin{array}{c} a_{i}\in \alpha_{1}\\ b_{i}\in \alpha_{2} \end{array}}{⋃}\max\left\{a_{i},b_{j}\right\}$

(ii)
$H_{1}\cap H_{2}=\underset{\begin{array}{c} a_{i}\in \alpha_{1}\\ b_{i}\in \alpha_{2} \end{array}}{⋃}\min\left\{a_{i},a_{j}\right\}$

(iii)
${H_{1}}^{c}=\underset{a_{i}\in \alpha_{1}}{⋃}\left\{{1-a}_{i}\right\}$

### 2.2. Aczel–Alsina Aggregation Operators

The realm of triangular norms (T.Ns), a distinct category of functions, offers a valuable framework for comprehending the convergence of fuzzy logic and fuzzy systems (FSs). These triangular norms find widespread employment across a spectrum of domains, notably in decision-making processes and data aggregation tasks. In the subsequent discussion, we delve into the pivotal concepts that play pivotal roles in the advancement of this discourse.

**Definition** **7** [45]**.** *A mapping* ∎: *[0, 1] × [0, 1] → [0, 1] is a called triangular norm by holding the given properties*,

∀ ∁,E, R *∈ [0, 1],*

(i) ∎ (∁ *,* E) = ∎ (E *,* ∁) *(Symmetrical)*
(ii) ∎ (∁, ∎ (E, R)) ∁ = ∎ (∎ (∁ *,* E), R) *(Associative)*
(iii) ∎ (∁ *,* E) ≤ ∎ (∁*,* R) if E ≤R *(Monotonic)*
(iv) ∎ (1, ∁) = ∁; *(One Identity)*

**Definition** **8** [45]**.** *A mapping* δ: *[0, 1] × [0, 1] → [0, 1] is a called triangular co-norm by holding the given properties*,

∀ ∁,E, R *∈ [0, 1],*

(i) δ (∁*,* E) = δ (E*,* ∁) *(Symmetrical)*
(ii) δ (∁, δ (E, R)) ∁ = δ (δ (∁ *,* E), R) *(Associative)*
(iii) δ (∁*,* E) ≤ δ (∁*,* R) if E ≤R *(Monotonic)*
(iv) δ (0, ∁) = ∁ ; *(Zero Identity)*

**Definition** **9** [45]**.** *Aczel–Alsina triangular norm and co-norms are defined and denoted as follows*,

∎′Aν∁, E=A′dra∁, E,      if ν=0min∁, E,      if ν=∞ e−(−log⁡∁ν+(−log E)ν)1ν,  otherwise

*and*

δ′Aν∁, E=B′dra∁, E,       if ν=0max∁, E       if ν=∞ 1−e−(−log⁡(1−∁)ν+(−log (1−E))ν)1ν,  otherwise

## 3. Development of the Model of Q-Rung Orthopair Hesitant Fuzzy Sets (Q-ROHFSs)

By amalgamating the concepts of Q-ROFSs and HFSs, we introduce an innovative framework named Q-ROHFSs, which exhibits an enhanced generality and exceptional efficacy in addressing the disparity between membership values and non-membership values. The idea of a Q-rung orthopair hesitant fuzzy set is as below:

**Definition** **10.** *Taking U as a ground set, A Q-rung orthopair hesitant fuzzy set* T*over U is formulated as below*.

$$T=\left(k,\gamma \left(k\right),\mu \left(k\right):k\epsilon U\right)$$

*where* γ *and*  μ *are a set of values chosen from the unit closed interval* $\left[0,1\right]$ *and denote the membership grade and non-membership grades, respectively, with satisfying the given condition.*

$$0\leq max\left(\gamma_{T}^{N}\left(k\right)\right)+max\left(\mu_{T}^{N}\left(k\right)\right)\leq 1,\;N\geq 1\;\forall \;k\;\epsilon \;U$$

*For ease of calculations, we will call* $T=\left(\gamma ,\mu \right)$ *a Q-ROHFN thoroughout the paper.*

**Definition** **11.** *For Q-ROHNs $T=\left(\gamma ,\mu \right),T_{1}=\left(\gamma_{1},\mu_{1}\right),$ and $T_{2}=\left(\gamma_{2},\mu_{2}\right)$, the following operators have been defined*.

-
$T_{1}⊕T_{2}=\underset{\begin{array}{c} a_{1}\in \gamma_{1}\\ a_{2}\in \gamma_{2}\\ b_{1}\in \mu_{1}\\ b_{2}\in \mu_{2} \end{array}}{⋃}\left(\sqrt[{N}]{1-\left(1-a_{1}^{N}\right)\left(1-a_{2}^{N}\right)},b_{1}b_{2}\right)$

-
$T_{1}⊗T_{2}=\underset{\begin{array}{c} a_{1}\in \gamma_{1}\\ a_{2}\in \gamma_{2}\\ b_{1}\in \mu_{1}\\ b_{2}\in \mu_{2} \end{array}}{⋃}\left(a_{1}a_{2},\sqrt[{N}]{1-\left(1-b_{1}^{N}\right)\left(1-b_{2}^{N}\right)}\right)$

-
$ℷT=\underset{\begin{array}{c} a_{1}\in \gamma_{1}\\ a_{2}\in \gamma_{2}\\ b_{1}\in \mu_{1}\\ b_{2}\in \mu_{2} \end{array}}{⋃}\left(\sqrt[{N}]{1-{\left(1-a^{N}\right)}^{ℷ}},b^{ℷ}\right)$

-
$T^{ℷ}=\underset{\begin{array}{c} a_{1}\in \gamma_{1}\\ a_{2}\in \gamma_{2}\\ b_{1}\in \mu_{1}\\ b_{2}\in \mu_{2} \end{array}}{⋃}\left(a^{ℷ},\sqrt[{N}]{1-{\left(1-b^{N}\right)}^{ℷ}}\right)$

-
${T_{1}}^{c}=\underset{\begin{array}{c} a_{1}\in \gamma_{1}\\ b_{1}\in \mu_{1} \end{array}}{⋃}\left(\left(b_{1},a_{1}\right)\right)$

**Definition** **12.** *The score function to rank the Q-ROHFNs* $T=\left(\gamma ,\mu \right)$ *is defined as*

(1) $$V\left(T\right)=\frac{S\left(\gamma \right)-S\left(\mu \right)}{2}$$

*For a Q-ROHFN, the accuracy function is designed as,*

$$W\left(T\right)=\frac{S\left(\gamma \right)+S\left(\mu \right)}{2}$$

*where* $S\left(\gamma \right)=\frac{Sum\;of\;elements\;in\;\gamma^{N}}{order\;of\;\gamma^{N}}$ and $S\left(\mu \right)=\frac{Sum\;of\;elements\;in\;\mu^{N}}{order\;of\;\mu^{N}}$

*It is evident that* $-1\leq V\left(T_{i}\right)\leq 1$ *and* $0\leq W\left(T_{i}\right)\leq 1.$

## 4. Q-Rung Orthopair Hesitant Fuzzy Aczel–Alsina (Q-ROHFAA) Aggregation Operators

In order to broaden and delve deeper into the applicability of Q-ROHFSs, we introduce several fundamental operational principles among Q-ROHFNs. Moreover, within this section, a set of Aczel–Alsina aggregation operators is introduced. This section encompasses the elucidation of the Aczel–Alsina operations concerning Q-ROHFSs, along with an exploration of the diverse fundamental properties inherent in these functions.

**Definition** **13.** *Let*$\;T_{1}=\left(\gamma_{1},\mu_{1}\right)$ *and $T_{2}=\left(\gamma_{2},\mu_{2}\right)$ be two Q-ROHFNs and $a_{1}\;\in \gamma_{T_{1}},a_{2}\;\in \gamma_{T_{2}},\;b_{1}\in \mu_{T_{1}},\;and\;b_{2}\in \mu_{T_{2}}$ with λ ≥ 1 and Θ > 0. Therefore, the basic operations for Q-ROFNs are designed as*:

-
$T_{1}⊕T_{2}=\underset{\begin{array}{c} a_{1}\in \gamma_{1}\\ a_{2}\in \gamma_{2}\\ b_{1}\in \mu_{1}\\ b_{2}\in \mu_{2} \end{array}}{⋃}\left(\sqrt[{N}]{\left(1-e^{-{\left({\left(-\log\left(1-a_{1}^{N}\right)\right)}^{\lambda }+{\left(-log\left(1-a_{2}^{N}\right)\right)}^{\lambda }\right)}^{\frac{1}{\lambda }}}\right)},\sqrt[{N}]{\left(e^{-{\left({\left(-log\left(b_{1}^{N}\right)\right)}^{\lambda }+{\left(-log\left(b_{2}^{N}\right)\right)}^{\lambda }\right)}^{\frac{1}{\lambda }}}\right)}\right)$

-
$T_{1}⊗T_{2}=\underset{\begin{array}{c} a_{1}\in \gamma_{1}\\ a_{2}\in \gamma_{2}\\ b_{1}\in \mu_{1}\\ b_{2}\in \mu_{2} \end{array}}{⋃}\left(\sqrt[{N}]{e^{-{\left({\left(-log\left(a_{1}^{N}\right)\right)}^{\lambda }+{\left(-log\left(a_{2}^{N}\right)\right)}^{\lambda }\right)}^{\frac{1}{\lambda }}}},\sqrt[{N}]{1-e^{-{\left({\left(-log\left(1-\left(b_{1}^{N}\right)\right)\right)}^{\lambda }+{\left(-log\left(1-\left(b_{2}^{N}\right)\right)\right)}^{\lambda }\right)}^{\frac{1}{\lambda }}}}\right)$

-
$\Theta T=\underset{\begin{array}{c} a\in \gamma \\ b\in \mu \end{array}}{⋃}\left(\sqrt[{N}]{1-e^{-{\left(\Theta {\left(-log\left(1-\left(a^{N}\right)\right)\right)}^{\lambda }\right)}^{\frac{1}{\lambda }}}},\sqrt[{N}]{e^{-{\left(\Theta {\left(-log\left(b^{N}\right)\right)}^{\lambda }\right)}^{\frac{1}{\lambda }}}}\right)$

-
$T^{\Theta }=\underset{\begin{array}{c} a\in \gamma \\ b\in \mu \end{array}}{⋃}\left(\sqrt[{N}]{e^{-{\left(\Theta {\left(-log\left(a^{N}\right)\right)}^{\lambda }\right)}^{\frac{1}{\lambda }}}},\sqrt[{N}]{1-e^{-{\left(\Theta {\left(-log\left(1-\left(b^{N}\right)\right)\right)}^{\lambda }\right)}^{\frac{1}{\lambda }}}}\right)$

**Theorem** **1.** *For two Q-ROHFNs,$\;T_{1}=\left(\gamma_{1},\mu_{1}\right)$ and $T_{2}=\left(\gamma_{2},\mu_{2}\right)$, with λ ≥ 1, Θ > 0. We have*

(i)
$T_{1}⊕T_{2}=T_{2}⊕T_{1}$

(ii)
$T_{1}⊗T_{2}=T_{2}⊗T_{1}$

(iii)
$\Theta (T_{1}⊕T_{2})={\Theta T}_{1}⊕\Theta T_{2}$

(iv)
${\left(T_{1}⊗T_{2}\right)}^{\Theta }=T_{1}^{\Theta }⊗T_{2}^{\Theta }$

(v)
$T^{\Theta_{1}}⊗T^{\Theta_{2}}=T^{\left(\Theta_{1}+\Theta_{2}\right)}$

**Proof** **.** Let $T,T_{1},$ and $T_{2}$ be Q-ROHFNs and $\Theta ,\Theta_{1},\;\Theta_{2}>0$, as revealed in Definition 14, we can calculate as,

(i)
$T_{1}⊕T_{2}$
$$=\underset{\begin{array}{c} a_{1}\in \gamma_{1}\\ a_{2}\in \gamma_{2}\\ b_{1}\in \mu_{1}\\ b_{2}\in \mu_{2} \end{array}}{⋃}\left(\sqrt[{N}]{1-e^{-{\left({\left(-\log\left(1-a_{1}^{N}\right)\right)}^{\lambda }+{\left(-log\left(1-\left(a_{2}^{N}\right)\right)\right)}^{\lambda }\right)}^{\frac{1}{\lambda }}}}\;,\;\sqrt[{N}]{e^{-{\left({\left(-log\left(b_{1}^{N}\right)\right)}^{\lambda }+{\left(-log\left(b_{2}^{N}\right)\right)}^{\lambda }\right)}^{\frac{1}{\lambda }}}}\right)\;=\underset{\begin{array}{c} a_{1}\in \gamma_{1}\\ a_{2}\in \gamma_{2}\\ b_{1}\in \mu_{1}\\ b_{2}\in \mu_{2} \end{array}}{⋃}\left(\sqrt[{N}]{1-e^{-{\left({\left(-\log\left(1-\left(a_{1}^{N}\right)\right)\right)}^{\lambda }+{\left(-log\left(1-\left(a_{1}^{N}\right)\right)\right)}^{\lambda }\right)}^{\frac{1}{\lambda }}}}\;,\;\sqrt[{N}]{e^{-{\left({\left(-log\left(b_{2}^{N}\right)\right)}^{\lambda }+{\left(-log\left(b_{1}^{N}\right)\right)}^{\lambda }\right)}^{\frac{1}{\lambda }}}}\right)\;=T_{2}⊕\;T_{1}$$
(ii) It is straightforward.
(iii)
$\Theta_{1}T⊕\;\Theta_{2}T$
$$=\underset{\begin{array}{c} a\in \gamma \\ b\in \mu \end{array}}{⋃}\left(\sqrt[{N}]{1-e^{-{\left(\Theta_{1}{\;\left(-log\left(1-\left(a^{N}\right)\right)\right)}^{\lambda }\right)}^{\frac{1}{\lambda }}}\;},\;\sqrt[{N}]{e^{-{\left(\Theta_{1}\;{\left(-log\left(b^{N}\right)\right)}^{\lambda }\right)}^{\frac{1}{\lambda }}}}\right)\;⊕\underset{\begin{array}{c} a\in \gamma \\ b\in \mu \end{array}}{⋃}\left(\sqrt[{N}]{1-e^{-{\left(\Theta_{2}{\;\left(-log\left(1-\left(a^{N}\right)\right)\right)}^{\lambda }\right)}^{\frac{1}{\lambda }}}\;},\;\sqrt[{N}]{e^{-{\left(\Theta_{2}\;{\left(-log\left(b^{N}\right)\right)}^{\lambda }\right)}^{\frac{1}{\lambda }}}}\right)\;=\underset{\begin{array}{c} a\in \gamma \\ b\in \mu \end{array}}{⋃}\left(\sqrt[{N}]{1-e^{-{\left((\Theta_{1}+\Theta_{2}){\;\left(-log\left(1-\left(a^{N}\right)\right)\right)}^{\lambda }\right)}^{\frac{1}{\lambda }}}}\;,\;\sqrt[{N}]{e^{-{\left((\Theta_{1}+\Theta_{2})\;{\left(-log\left(b^{N}\right)\right)}^{\lambda }\right)}^{\frac{1}{\lambda }}}}\right)\;={(\Theta }_{1}+\Theta_{2})T$$
(iv)
${\left(T_{1}⊗T_{2}\right)}^{\Theta }$
$$=\underset{\begin{array}{c} a_{1}\in \gamma_{1}\\ a_{2}\in \gamma_{2}\\ b_{1}\in \mu_{1}\\ b_{2}\in \mu_{2} \end{array}}{⋃}\left(\sqrt[{N}]{{\left(e^{-{\left({\left(-log\left(a_{1}^{N}\right)\right)}^{\lambda }+{\left(-log\left(a_{2}^{N}\right)\right)}^{\lambda }\right)}^{\frac{1}{\lambda }}}\right)}^{\Theta }},\sqrt[{N}]{{\left(1-e^{-{\left({\left(-log\left(1-\left(b_{1}^{N}\right)\right)\right)}^{\lambda }+{\left(-log\left(1-\left(b_{2}^{N}\right)\right)\right)}^{\lambda }\right)}^{\frac{1}{\lambda }}}\right)}^{\Theta }\;}\right)\;=\underset{\begin{array}{c} a_{1}\in \gamma_{1}\\ a_{2}\in \gamma_{2}\\ b_{1}\in \mu_{1}\\ b_{2}\in \mu_{2} \end{array}}{⋃}\left(\sqrt[{N}]{e^{-{\left(\Theta {\left(-log\left(a_{1}^{N}\right)\right)}^{\lambda }+{\left(-log\left(a_{2}^{N}\right)\right)}^{\lambda }\right)}^{\frac{1}{\lambda }}}},\sqrt[{N}]{1-e^{-{\left(\Theta {\left(-log\left(1-\left(b_{1}^{N}\right)\right)\right)}^{\lambda }+{\left(-log\left(1-\left(b_{2}^{N}\right)\right)\right)}^{\lambda }\right)}^{\frac{1}{\lambda }}}}\right)\;=\underset{\begin{array}{c} a_{1}\in \gamma_{1}\\ a_{2}\in \gamma_{2}\\ b_{1}\in \mu_{1}\\ b_{2}\in \mu_{2} \end{array}}{⋃}\left(\sqrt[{N}]{e^{-{\left(\Theta {\left(-log\left(a_{1}^{N}\right)\right)}^{\lambda }\right)}^{\frac{1}{\lambda }}}},\sqrt[{N}]{1-e^{-{\left(\Theta {\left(-log\left(1-\left(b_{1}^{N}\right)\right)\right)}^{\lambda }\right)}^{\frac{1}{\lambda }}}}\right)\;⊗\underset{\begin{array}{c} a_{1}\in \gamma_{1}\\ a_{2}\in \gamma_{2}\\ b_{1}\in \mu_{1}\\ b_{2}\in \mu_{2} \end{array}}{⋃}\left(\sqrt[{N}]{e^{-{\left(\Theta \;{\left(-log\left(a_{2}^{N}\right)\right)}^{\lambda }\right)}^{\frac{1}{\lambda }}}},\sqrt[{N}]{1-e^{-{\left(\Theta \;{\left(-log\left(1-\left(b_{2}^{N}\right)\right)\right)}^{\lambda }\right)}^{\frac{1}{\lambda }}}}\right)\;=T_{1}^{\Theta }⊗T_{2}^{\Theta }$$
(v)
$T^{\Theta_{1}}⊗T^{\Theta_{2}}$
$$=\underset{\begin{array}{c} a\in \gamma \\ b\in \mu \end{array}}{⋃}\left(\sqrt[{N}]{e^{-{\left(\Theta_{1}\;{\left(-log\left(a^{N}\right)\right)}^{\lambda }\right)}^{\frac{1}{\lambda }}}},\;\sqrt[{N}]{1-e^{-{\left(\Theta_{1}{\;\left(-log\left(1-\left(b^{N}\right)\right)\right)}^{\lambda }\right)}^{\frac{1}{\lambda }}}}\right)\;⊗\underset{\begin{array}{c} a\in \gamma \\ b\in \mu \end{array}}{⋃}\left(\sqrt[{N}]{e^{-{\left(\Theta_{2}\;{\left(-log\left(a^{N}\right)\right)}^{\lambda }\right)}^{\frac{1}{\lambda }}}},\;\;\sqrt[{N}]{1-e^{-{\left(\Theta_{2}{\;\left(-log\left(1-\left(b^{N}\right)\right)\right)}^{\lambda }\right)}^{\frac{1}{\lambda }}}}\right)\;=\underset{\begin{array}{c} a\in \gamma \\ b\in \mu \end{array}}{⋃}\left(\sqrt[{N}]{e^{-{\left((\Theta_{1}+\Theta_{2})\;{\left(-log\left(a^{N}\right)\right)}^{\lambda }\right)}^{\frac{1}{\lambda }}}},\;\sqrt[{N}]{1-e^{-{\left((\Theta_{1}+\Theta_{2}){\;\left(-log\left(1-\left(b^{N}\right)\right)\right)}^{\lambda }\right)}^{\frac{1}{\lambda }}}}\right)\;=T^{\left(\Theta_{1}+\Theta_{2}\right)}□$$

### Q-Rung Orthopair Hesitant Fuzzy Aczel–Alsine Average (Q-ROHFAAA) Aggregation Operators

Here, some Q-ROHFAA average aggregation operators and their properties are discussed in detail.

**Definition** **14.** *Consider* $T_{i}=\left(\gamma_{i},\;\mu_{i}\;\right),(i=1,2,\ldots ,n)$ *as Q-ROHFNs and their corresponding weight $\delta ={\left(\delta_{1},\delta_{2},\ldots {,\delta }_{n}\right)}^{T}$ for the $T_{i}$ with $\delta_{i}$ > 0, $\delta_{i}\in \left[0,1\right]$ and $\sum_{i=1}^{n}\delta_{i}=1.\;$Then the Q-ROHFAAWA operator is a function: Q-ROHFAAWA*: ${\left(T\right)}^{n}\to T$*defined as*

Q-ROHFAAWA T1,T2…,Tn=⊕i=1nδiTi=δ1T1⊕δ2T2⊕,…,⊕δnTn=⋃ai∈γibi∈μi1−e−∑i=1nδi−log⁡1−aiNλ1λN,e−∑i=1nδi−log⁡(biN)λ1λN 

Definition 14 is directed to establishing Theorem 2.

**Theorem** **2.** *Suppose $T_{i}=\left(\gamma_{i},\;\mu_{i}\;\right)$ is a set of Q-ROHFNs. The assigned weight δ for each $T_{i}.$ The gained product of Q-ROHFNs applying the Q-ROHFAAWA operator is again Q-ROHFN*:

(2) Q-ROHFAAWA T1,T2,…,Tn=⊕i=1nδiTi=⋃ai∈γibi∈μi1−e−∑i=1nδi−log⁡1−aiNλ1λN,e−∑i=1nδi−log⁡(biN)λ1λN 

**Proof** **.** The mathematical induction technique helps to proof the above result.

(I) Let i=2, then

δ1T1=⋃a1∈γ1b1∈μ11−e−δ1−log⁡1−a1Nλ1λN,e−δ1−log⁡(b1N)λ1λN δ2T2=⋃a2∈γ2b2∈μ21−e−δ2−log⁡1−a2Nλ1λN,e−δ2−log⁡(b2N)λ1λN  

We obtain the following result using Definition 15,

(3) Q-ROHFAAWA T1,T2=δ1T1⊕δ2T2=⋃a1∈γ1b1∈μ11−e−δ1−log⁡1−a1Nλ1λN,e−δ1−log⁡(b1N)λ1λN ⊕⋃a2∈γ2b2∈μ21−e−δ2−log⁡1−a2Nλ1λN,e−δ2−log⁡(b2N)λ1λN =⋃a1∈γ1a2∈γ2b1∈μ1b2∈μ21−e−δ1−log⁡1−a1Nλ+δ2−log⁡1−a2Nλ1λN,e−δ1−log⁡(b1N)λ+δ2−log⁡(b2N)λ1λN=⋃a1∈γ1a2∈γ2b1∈μ1b2∈μ21−e−∑i=12δi−log⁡1−aiNλ1λN,e−∑i=12δi−log⁡(biN)λ1λN

Hence, Equation (3) is fulfilled for i=2.

(II) Taking into consideration Equation (3) is fulfilled for *i* = *k*, then the following is obtained

Q-ROHFAAWA T1,T2,…,Tk=⊕i=1kδiTi=⋃ai∈γibi∈μi1−e−∑i=1kδi−log⁡1−aiNλ1λN,e−∑i=1kδi−log⁡(biN)λ1λN 

Now, for i=k+1, we obtain,

(4) Q-ROHFAAWAT1,T2,…,Tk,Tk+1=⊕s=1kδiTi⊕δk+1Tk+1=⋃ai∈γibi∈μi1−e−∑i=1kδi−log⁡1−aiNλ1λN,e−∑i=1kδi−log⁡(biN)λ1λN ⊕⋃ai∈γk+1bi∈μk+11−e−δk+1(−log⁡1−ak+1Nλ)1λN,e−δk+1−log⁡(bk+1N)λ1λN =⋃ai∈γibi∈μi1−e−∑i=1k+1δi(−log⁡1−aiNλ)1λN,e−∑i=1k+1δi−log⁡(biN)λ1λN 

Thus, Equation (4) is legitimate for i=k+1.

(I), (II) indicates that it can be deduced that Equation (4) is fulfilled for any i. Hence, it is proved. □

From a Q-ROHFAAWA aggregation operator, the following properties (Idempotency, Boundedness, and Monotonicity) can be illustrated.

**Property** **1.** *If* $T_{i}=\left(\gamma_{i},\;\mu_{i}\;\right),\forall i\;$*are similar*,

$$T_{i}=T\forall i,\;then\;Q-ROHFAAWA\;\left(T_{1},T_{2},\ldots ,T_{i}\right)=T.$$

**Property** **2.** *Consider* $T_{i}=\left(\gamma_{i},\;\mu_{i}\;\right),$ *are Q-ROHFNs. if* $T^{-}=inf(T_{1,}T_{2,}\ldots ,T_{n})\;$ *and* $T^{+}=sup(T_{1,}T_{2,}\ldots ,T_{n})$. *Then*,

$$T^{-}\leq Q-ROHFAAWA\left(T_{1,}T_{2,}\ldots ,T_{n}\right)\leq T^{+}.$$

**Property** **3.** *For* $T_{i}\;$ *and* $\;T_{i}^{\prime }$, *where* $T_{i}\leq T_{i}^{\prime }\;\forall \;i$ *then Q-ROHFAAWA* $\left(T_{1,}T_{2,}\ldots ,T_{n}\right)\leq Q-ROHFAAWA\left(T_{1,}^{\prime }T_{2,}^{\prime }\ldots ,T_{n}^{\prime }\right)$.

Currently, we produce Q-ROHF Aczel–Alsina ordered weighted averaging Q-ROHFAAWA operations.

**Definition** **15.** *Suppose* $T_{i}=\left(\gamma_{i},\;\mu_{i}\;\right)$ *is a collection of Q-ROHFNs and the assigned weight* $\delta ={\left(\delta_{1},\delta_{2},\ldots {,\delta }_{n}\right)}^{T}$ *for all* $T_{i}$ *and* $\sum_{i=1}^{n}\delta_{i}=1.$ *Then, the Q-ROHFAAOWA operator is a mapping, such that Q-ROHFAAOWA*: $T^{n}\to T$*defined as*,

Q-ROHFAAOWA T1,T2…,Tn=⊕s=1nδiTσi=δ1Tσ1⊕δ2Tσ2⊕,…,⊕δnTσn=⋃ai∈γibi∈μi1−e−∑i=1nδi(−log⁡1−aσiNλ 1λN,e−∑i=1nδi−log⁡(bσiN)λ1λN 

*where* ($\sigma \left(1\right),\sigma \left(2\right),\ldots ,\sigma (n)$) *are the permutations of* ∀i*, enclosing* $T_{\sigma (n-1)}\geq T_{\sigma (n)}$.

From Definition 15, we obtain the result shown below.

**Theorem** **3.** *Assume* $T_{i}=\left(\gamma_{i},\;\mu_{i}\;\right)$ *is a collection of Q-ROHFNs. The allocated weight * δ. *The calculated number of Q-ROHFNs by the Q-ROHFAAOWA aggregation operator is also Q-ROHFN*:

Q-ROHFAAOWAT1,T2,…,Tn=⊕i=1nδiTσi=⋃ai∈γibi∈μi1−e−∑i=1nδi(−log⁡1−aσiNλ 1λN,e−∑i=1nδi−log⁡(bσiN)λ1λN 

*where* ($\sigma \left(1\right),\sigma \left(2\right),\ldots ,\sigma (n)$) *are the permutations of every* i, *containing* $T_{\sigma (n-1)}\geq T_{\sigma (n)}$.

From the Q-ROHFAAOWA aggregation operator, the following properties (Idempotency, Boundedness, Monotonicity, and Commutativity) can be easily illustrated.

**Property** **4.** *Let* $T_{i}=\left(\gamma_{i},\;\mu_{i}\;\right),(i=1,2,\ldots ,n)$ *be the same, there is*,

$$T_{i}=T\forall i,\;\mathrm{then}Q-\mathrm{ROHFAAOWA}\left(T_{1},T_{2},\ldots ,T_{i}\right)=T.$$

**Property** **5.** *If all* $T_{i}=\left(\gamma_{i},\;\mu_{i}\;\right)$ *are sets of Q-ROHFNs, consider* $T^{-}=inf(T_{1,}T_{2,}\ldots ,T_{n})\;$ and $T^{+}=sup(T_{1,}T_{2,}\ldots ,T_{n})$. *We have*,

$$T^{-}\leq Q-ROHFAAOWA\left(T_{1,}T_{2,}\ldots ,T_{n}\right)\leq T^{+}.$$

**Property** **6.** *Let* $T_{i}\leq T_{i}^{\prime }\;\forall \;i$ *then*,

$$Q-\mathrm{ROHFAAOWA}\;\left(T_{1,}T_{2,}\ldots ,T_{n}\right)\leq Q-ROHFAAOWA\left(T_{1,}^{\prime }T_{2,}^{\prime }\ldots ,T_{n}^{\prime }\right).$$

**Property** **7.** *Let* $T_{i}\;$ and $\;T_{i}^{\prime }$ *be a collection of Q-ROHFNs, then* $Q-ROHFAAOWA\left(T_{1,}T_{2,}\ldots ,T_{n}\right)=Q-ROHFAAOWA\left(T_{1,}^{\prime }T_{2,}^{\prime }\ldots ,T_{n}^{\prime }\right),$ *where* $T_{i}^{\prime }$ *is any permutation of *$T_{i}\;$.

Definition 14 and Definition 15 provide guidance to develop hybrid aggregation operators, which is stated below.

**Definition** **16.** *Suppose* $T_{i}=\left(\gamma_{i},\;\mu_{i}\;\right),$ *and the allotted weight* $\delta ={\left(\delta_{1},\delta_{2},\ldots {,\delta }_{n}\right)}^{T}$ *for every* $T_{i}$ *and a new* ${\dot{T}}_{i}=n\delta_{i}T_{i}.$ *Then the Q-ROHFAAHWA operator is a mapping Q-ROHFAAHWA*: ${\dot{T}}^{n}\to \dot{T}$*defined as*

Q-ROHFAAHW T˙1,T˙2…,T˙n=⊕i=1nδiT˙σi=δ1T˙σ1⊕δ2T˙σ2⊕,…,⊕δnT˙σn=⋃a˙i∈γ˙ib˙i∈μ˙i1−e−∑i=1nδi(−log⁡1−a˙σiNλ1λN,e−∑i=1nδi−log⁡(b˙σiN)λ1λN 

*where* ($\sigma \left(i\right)$) *denotes the permutations of all* i, *containing* ${\dot{T}}_{\sigma (n-1)}\geq {\dot{T}}_{\sigma (n)}$.

Definition 16 is capable of promoting the concept which is presented in Theorem 4.

**Theorem** **4.** *For Q-ROHFNs, $T_{i}=\left(\gamma_{i},\;\mu_{i}\;\right)$. The result using the Q-ROHFAAHWA aggregation operator for Q-ROHFNs is still a Q-ROHFN.*

Q-ROHFAAHWA T1,T2,…,Tn=⊕i=1nδiT˙σi=⋃ai∈γibi∈μi1−e−∑i=1nδi(−log⁡1−aσiNλ1λN, e−∑i=1nδi−log⁡(bσiN)λ1λN 

**Proof**  The proof is skipped. □

**Theorem** **5.** *The Q-ROHFAAHWA aggregation operators are a simplification of the Q-ROHFAAWA and Q-ROHFAAOWA operators*.

**Proof.** 

(1) Let $\delta ={\left(\frac{1}{n},\frac{1}{n},\ldots \frac{1}{n}\right)}^{T}$ Then

$$\begin{array}{cc} Q-\mathrm{ROHFAAHWA}\;(T_{1},T_{2},\ldots ,T_{n}) & \\ & =\delta_{1}{\dot{T}}_{\sigma \left(1\right)}⊕\delta_{2}{\dot{T}}_{\sigma \left(2\right)}⊕\ldots ,⊕\delta_{n}{\dot{T}}_{\sigma \left(n\right)}\\ & =\frac{1}{n}\left(\delta_{1}{\dot{T}}_{\sigma \left(1\right)}⊕{\dot{T}}_{\sigma \left(2\right)}⊕\ldots ,⊕{\dot{T}}_{\sigma \left(n\right)}\right)\\ & =\delta_{1}T_{\sigma \left(1\right)}⊕\delta_{2}T_{\sigma \left(2\right)}⊕\ldots ,⊕\delta_{n}T_{\sigma \left(n\right)}\\ =Q-\mathrm{ROHFAAWA}\;(T_{1},T_{2},\ldots ,T_{n}) & \end{array}$$

(2) Let $\delta =\left(\frac{1}{n},\frac{1}{n},\ldots \frac{1}{n}\right)$ Then

$$Q-ROHFA_{A}HA_{\delta }\left(T_{1},T_{2},\ldots ,T_{n}\right)\;=\delta_{1}{\dot{T}}_{\sigma \left(1\right)}⊕\delta_{2}{\dot{T}}_{\sigma \left(2\right)}⊕\ldots ,⊕\delta_{n}{\dot{T}}_{\sigma \left(n\right)}\;=\delta_{1}T_{\sigma \left(1\right)}⊕\delta_{2}T_{\sigma \left(2\right)}⊕\ldots ,⊕\delta_{n}T_{\sigma \left(n\right)}\;=Q-\mathrm{ROHFAAOWA}\;\left(T_{1},T_{2},\ldots ,T_{n}\right)$$

which completes the proof. □

## 5. Proposed Decision-Making Approach Based on Q-Rung Orthopair Hesitant Fuzzy Aczel–Alsina Aggregation Operators

**Figure 2:**
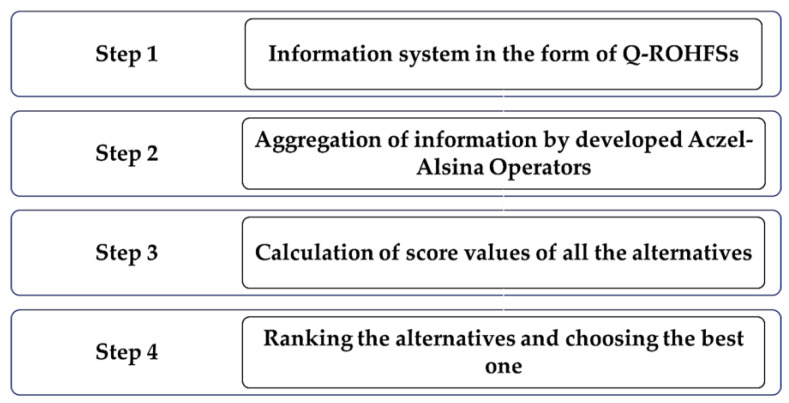
Representation of the algorithm.

This section is established based on the solution for the decision-making challenge under the Q-ROHFSs, and we apply the established Q-ROHFAA aggregation operators. The decision-making algorithm is premeditated by the following notations:

Let $T=\left\{T_{1},T_{2},\ldots ,T_{m}\right\}$ be set of m numerous alternatives, which must be observed under the gathering of $\prime n^{\prime }$ numerous criteria $B_{i}=\left\{B_{1},B_{2},\ldots ,B_{n}\right\}.$ Suppose that these alternatives are scrutinized using an expert who states their partialities in relation to each alternative $T_{i}\left(i\in m\right)$ for Q-ROHF information, and these digits may be recognized as Q-ROHFSs $D={\left(T_{ij}\right)}_{m\times n}$, such that $T_{ij}=\left(\gamma_{ij},\mu_{ij}\right)$ shows the priority values of an alternative $B_{i}$ given by a decision maker. Let $w={\left(w_{1},w_{2},\ldots ,w_{n}\right)}^{T}\;b$e the weight i vector of the criteria i, such that $w_{i}>0$ and $\sum_{i=1}^{n}w_{i}=1.$ The recommended policy is separated into the following steps in order to decide the best alternative(s), and Figure 2 signifies the procedure step by step.

**Step 1:** Obtain information on the alternative ratings that relate to conditions and express it in the system of Q-ROHFS $T_{ij}=\left(\gamma_{ij},\mu_{ij}\right):i=1,2i,\ldots ,im;j=1,i2,\ldots ,n$. These rating i results are stated as a decision matrix D as

D=..T1T2⋮Tm B1B2⋯BnT11T21T12T22⋯T12T2n⋮⋱⋱⋮Tm1Tm2⋯Tmn

**Step 2**: Aggregate the various preference results $T_{ij},\;j=1,2,\ldots ,n$ of the alternatives $B_{i}$ into the collective one $T_{i},\;$using Q-ROHFHWA aggregation operators as
  Q-ROHFAAHWA Tij=⋃ai∈γibi∈μi1−e−∑i=1nδi(−log⁡1−aσiNλ1λN,e−∑i=1nδi−log⁡(bσiN)λ1λN 

**Step 3:** Aggregate the score i value of the aggregated Q-ROHFNs $T_{i}$, applying the given formula,
  $$V\left(T\right)=\frac{S\left(\gamma \right)-S\left(\mu \right)}{2}$$

**Step 4:** Select the best alternative based on the score values.

### 5.1. Mathematical Model

To demonstrate the realistic use of the recommended approach, a mathematical model is given below.

Wireless Sensor Networks (WSNs) are networks composed of a large number of tiny, low-cost, autonomous devices called sensor nodes. These nodes are equipped with various types of sensors to collect and transmit data from their surrounding environment. WSNs are commonly used in a wide range of applications, including environmental monitoring, surveillance, healthcare, industrial automation, and more.

A company is deploying a process for selecting the best gateway node in a Wireless Sensor Network (WSN) to ensure reliable communication and data aggregation. The company has identified four potential gateway nodes $T=\{T_{1},T_{2},T_{3},T_{4}\;\}$, each with its own set of attributes $B=\left\{DBS,BV,DAE,SNR\right\}$ that are important to the decision. However, because the attributes are uncertain and imprecise, traditional crisp values may not accurately reflect real-world conditions. The attributes are given as **Distance to Base Station (DBS):** the distance between each potential gateway node and the base station. Smaller distances are preferred for better communication reliability. **Battery Voltage (BV):** the voltage level of the battery in each potential gateway node. A higher battery voltage implies more energy and a longer network lifetime. **Data Aggregation Efficiency (DAE):** the efficiency of each gateway node in aggregating data from other sensor nodes. A higher efficiency leads to better data collection and reduced network congestion. **Signal-to-Noise Ratio (SNR):** the signal-to-noise ratio of the communication link for each gateway node. Higher SNR indicates a better communication quality. Using Q-ROHFAAWA aggregation operators, we will evaluate the given uncertain data of all the alternatives and then select the best gateway node for a WSN.

Step 1: The Q-ROHF data are provided by the expert in Table 2.

**Table 2:** Shows the Q-ROHFNs.

Alternatives	DBS	BV	DAE	SNR
$T_{1}$	$\left\{0.8,0.3\right\},\{0.2,0.1\}$	$\left\{0.3,0.2\right\},\{0.1,0.5\}$	$\left\{0.1,0.3\right\},\{0.2,0.5\}$	$\left\{0.5,0.4\right\},\{0.3,0.1\}$
$T_{2}$	$\left\{0.4,0.3\right\},\{0.3,0.5\}$	$\left\{0.1,0.6\right\},\{0.2,0.3\}$	$\left\{0.3,0.1\right\},\{0.4,0.5\}$	$\left\{0.4,0.2\right\},\{0.1,0.3\}$
$T_{3}$	$\left\{0.4,0.2\right\},\{0.4,0.1\}$	$\left\{0.5,0.3\right\},\{0.4,0.1\}$	$\left\{0.4,0.5\right\},\{0.1,0.0\}$	$\left\{0.5,0.6\right\},\{0.2,0.2\}$
$T_{4}$	$\left\{0.2,0.6\right\},\{0.3,0.4\}$	$\left\{0.3,0.4\right\},\{0.2,0.5\}$	$\left\{0.5,0.3\right\},\{0.2,0.3\}$	$\left\{0.4,0.5\right\},\{0.2,0.1\}$

Step 2: The Q-ROHF information is calculated by the proposed Q-ROHFAAWA aggregation operator. For the above-mentioned information, the weight vectors are $w={\left\{0.35,0.25,0.25,0.15\right\}}^{T}$. The information is aggregated by considering the parameter N = 1 and λ=1 and displayed in Table 3.

**Table 3:** Aggregated results of alternative by the Aczel–Alsina operator.

Alternatives	Q-ROHFAAHWA
$T_{1}$	$\left\{0.5428,0.2927\right\},\{0.1787,0.2236\}$
$T_{2}$	$\left\{0.3099,0.3388\right\},\{0.2470,0.4075\}$
$T_{3}$	$\left\{0.4421,0.3799\right\},\{0.2549,0.1109\}$
$T_{4}$	$\left\{0.3419,0.4735\right\},\{0.2304,0.3197\}$

Step 3: In this stage, the calculation of the membership grades and non-membership grades is performed using score values, and the best alternative is ranked below,

$$T_{3}>T_{1}>T_{4}>T_{2}$$

From the above ranking, it is concluded that the $T_{3}$ is the best gateway node.

### 5.2. Analysis of Parameters on Decision Making

In this section, we will analyze the impact of the Q-ROHFN parameter N and the Aczel–Alsina aggregation operators λ. Table 4 shows the scores of all the alternatives obtained during the parameter alternation. Table 5 displays the ranking results based on the score values calculated in the given table.

**Figure 3:**
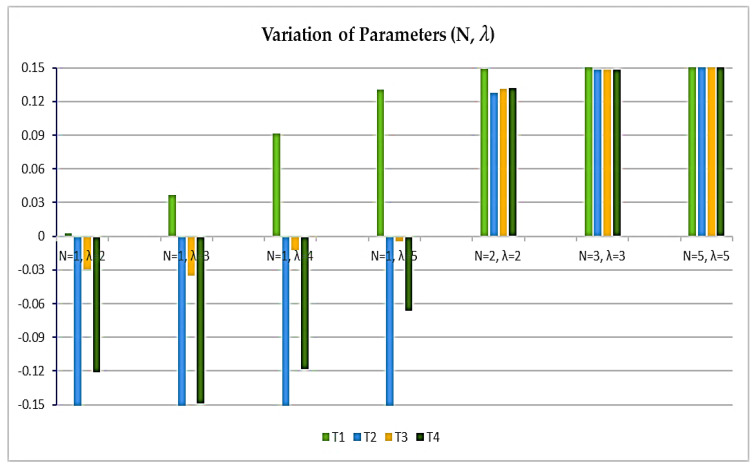
Geometrical representation of parameters.

Figure 3 provides a visual representation illustrating how changes in the parameters impact the system. This visual aid aids in grasping the concept of parameter variation’s influence on the alternatives. The noteworthy insight gleaned from this graph is that, as the fuzzy parameter N increases, it augments the positive attributes of the alternatives, ultimately leading to a convergence of their values.

Table 5 shows the rankings of the alternatives on the basis of the score values with the change in parameters.

### 5.3. Discussion

In this section, a detailed analysis of the proposed model is discussed. Based on the information provided in Table 5, it is apparent that, when N = 1 and λ takes values of 1, 2, 3, 4, and 5, the ranking of the alternatives remains consistent, with $T_{1}$ emerging as the optimal choice in this context. However, as we vary the Q-ROHFN parameter, considering values of N = 2, 3, and 5, the ranking of the alternatives experiences a gradual transformation. Ultimately, when N assumes these values, we observe that $T_{2},T_{3},and\;T_{4}$ yield identical scores, while $T_{1}$ continues to be the most suitable gate node. It is evident that, with an increasing N, the alternatives’ attributes lead to comparable score values, highlighting the influence of N on the ranking outcomes.

### 5.4. Benefits of the Established Approach

The decision-making model introduced in this paper is both highly attractive and exceptionally effective in addressing the challenges posed by uncertainty. The advantages of this established model are elaborated upon below:

- The newly developed model, known as the Q-ROHF model, stands out as a more comprehensive fuzzy model. It achieves this by amalgamating the Q-ROF model framework with the IHF model, resulting in a powerful and versatile tool.
- The Q-ROHF model has the unique ability to encompass both HF models and Q-ROF models simultaneously, enhancing its applicability and versatility.
- By manipulating the fuzzy parameter N, we can derive results applicable to intuitionistic hesitant fuzzy sets, Pythagorean hesitant fuzzy sets, and Fermatean hesitant fuzzy sets. This adaptability allows the model to cater to a wide range of scenarios.
- The proposed approach further extends its generality by encompassing Aczel–Alsina aggregation operators. This includes operators such as Q-ROHFAAWA, Q-ROHFAAOWA, and Q-ROHFAAHWA, which are instrumental to calculating information in various contexts.
- The model’s flexibility is further underscored by its ability to accommodate a variety of fuzzy and Aczel–Alsina parameters. This flexibility enables the exploration of different parameter values and their respective effects on alternatives and their rankings, making it a valuable tool for decision makers.

## 6. Conclusions

There was a gap in the literature about the development of Aczel-Alsina aggregation operators, which are the most efficient and popular aggregation operators for Q-ROHFS environments. In this paper, we introduced an innovative and robust fuzzy model known as Q-ROHFSs, specifically designed to address the challenges posed by uncertain data in our contemporary world. This novel approach significantly enhanced our ability to tackle uncertainty-related issues and proved invaluable for decision-making models. Our research explored various facets of fuzzy decision-making models and demonstrated their practical applicability within Wireless Sensor Networks (WSNs). Furthermore, we seamlessly integrated two distinct fuzzy models, Q-ROFSs and HFSs, and provided a comprehensive analysis of their unique characteristics. We delved deeply into the foundational properties of our proposed model, thoroughly explaining concepts such as monotonicity, commutativity, and boundedness. Within the framework of Q-ROHFSs, we introduced a series of Aczel–Alsina aggregation operators, including Q-ROHFAAWA, Q-ROHFAAOWA, and Q-ROHFAAHWA, to enhance the versatility of our approach. These operators were rigorously examined, confirming their ability to maintain the essential properties of Q-ROHFN. Moreover, we developed a mathematical model tailored for multiple-attribute decision making in the context of WSNs, founded on our established methodology. To provide a clearer understanding of our findings, we conducted a concise analysis of how parameter variations impacted alternative rankings, complemented by graphical representations illustrating these ranking fluctuations. Despite the promising contributions of our research, there are certain limitations and avenues for future research that should be considered. Firstly, while our proposed Q-ROHFSs model demonstrated its potential for handling uncertain data, further empirical validation and real-world application studies are needed to assess its performance in diverse practical scenarios. Additionally, the integration of Q-ROHFSs with Aczel–Alsina aggregation operators, while enhancing versatility, may require a more in-depth exploration of their properties and the development of additional operators to cater to specific decision-making contexts. Furthermore, our focus on Wireless Sensor Networks (WSNs) primarily centered on selecting optimal gateway nodes, leaving room for investigating the other decision-making aspects within WSNs.

Future research could explore the scalability and computational complexity of our proposed model in large-scale WSNs and investigate its applicability to various decision tasks in this domain. Finally, addressing the interpretability and user friendliness of the Q-ROHFSs model, especially in practical decision support systems, represents a valuable direction for further research, potentially involving human-centric interfaces and visualization techniques. The existing models in [53,54,55,56] will be expanded using the suggested methodology.

## Figures and Tables

**Table 1:** Symbols and descriptions.

Symbols	Description	Symbols	Description
Q-ROFSs	Q-rung orthopair fuzzy sets	Q-ROHFAAWA	Q-rung orthopair hesitant fuzzy Aczel–Alsina Weighted Average
HFSs	Hesitant fuzzy sets	Q-ROHFAAOWA	Q-rung orthopair hesitant fuzzy Aczel–Alsina Ordered Weighted Average
Q-ROHFSs	Q-rung orthopair hesitant fuzzy sets	Q-ROHFAAHWA	Q-rung orthopair hesitant fuzzy Aczel–Alsina Hybrid Weighted Average
Q-ROHFNs	Q-rung orthopair hesitant fuzzy numbers	WSNs	Wireless Sensor Networks

**Table 4:** Score results by the variation of parameters.

Parameters		Alternatives	Score Values
N = 1 and λ=2		$T_{1}$	0.0028
		$T_{2}$	−0.1960
		$T_{3}$	−0.0297
		$T_{4}$	−0.1211
N = 1 and λ=3		$T_{1}$	0.0366
		$T_{2}$	−0.2353
		$T_{3}$	−0.0349
		$T_{4}$	−0.1488
N = 1 and λ=4		$T_{1}$	0.0918
		$T_{2}$	−0.2278
		$T_{3}$	−0.0121
		$T_{4}$	−0.118
N = 1 and λ=5		$T_{1}$	0.1307
		$T_{2}$	−0.208
		$T_{3}$	−0.0044
		$T_{4}$	−0.0664
N = 2 and λ=2		$T_{1}$	0.1491
		$T_{2}$	0.1274
		$T_{3}$	0.1315
		$T_{4}$	0.1323
N = 3 and λ=3		$T_{1}$	0.1529
		$T_{2}$	0.1482
		$T_{3}$	0.1483
		$T_{4}$	0.1484
N = 5 and λ=5		$T_{1}$	0.1639
		$T_{2}$	0.1638
		$T_{3}$	0.1638
		$T_{4}$	0.1638

**Table 5:** Rankings by the variation of parameters.

Parameters	Ranking
N = 1 and λ=2	$T_{1}>T_{3}>T_{4}>T_{2}$
N = 1 and λ=3	$T_{1}>T_{3}>T_{4}>T_{2}$
N = 1 and λ=4	$T_{1}>T_{3}>T_{4}>T_{2}$
N = 1 and λ=5	$T_{1}>T_{3}>T_{4}>T_{2}$
N = 2 and λ=2	$T_{1}>T_{4}>T_{3}>T_{2}$
N = 3 and λ=3	$T_{1}>T_{4}>T_{3}>T_{2}$
N = 5 and λ=5	$T_{1}>T_{4}>T_{4}>T_{4}$

## Data Availability

Not applicable.
